# True malaria prevalence in children under five: Bayesian estimation using data of malaria household surveys from three sub-Saharan countries

**DOI:** 10.1186/s12936-018-2211-y

**Published:** 2018-02-05

**Authors:** Elvire Mfueni, Brecht Devleesschauwer, Angel Rosas-Aguirre, Carine Van Malderen, Patrick T. Brandt, Bernhards Ogutu, Robert W. Snow, Léon Tshilolo, Dejan Zurovac, Dieter Vanderelst, Niko Speybroeck

**Affiliations:** 1 Institute of Health and Society, Université Catholique de Louvain, Brussels, Belgium; 2 Department of Public Health and Surveillance, Scientific Institute of Public Health (WIV-ISP), Brussels, Belgium; 3 School of Economic, Political and Policy Sciences, The University of Texas, Dallas, TX USA; 4 Kenya Medical Research Institute, Kisumu, Kenya; 5 Population & Health Theme, Kenya Medical Research Institute/Wellcome Trust Research Programme, Nairobi, Kenya; 6 Centre for Tropical Medicine and Global Health, Nuffield Department of Clinical Medicine, University of Oxford, Oxford, UK; 7 Centre Hospitalier Monkole, Kinshasa, Democratic Republic of the Congo; 8 Department of Biology, University of Cincinnati, Cincinnati, OH USA

**Keywords:** Bayesian data analysis, Malaria, Sub-Saharan Africa, True prevalence

## Abstract

**Background:**

Malaria is one of the major causes of childhood death in sub-Saharan countries. A reliable estimation of malaria prevalence is important to guide and monitor progress toward control and elimination. The aim of the study was to estimate the true prevalence of malaria in children under five in the Democratic Republic of the Congo, Uganda and Kenya, using a Bayesian modelling framework that combined in a novel way malaria data from national household surveys with external information about the sensitivity and specificity of the malaria diagnostic methods used in those surveys—i.e., rapid diagnostic tests and light microscopy.

**Methods:**

Data were used from the Demographic and Health Surveys (DHS) and Malaria Indicator Surveys (MIS) conducted in the Democratic Republic of the Congo (DHS 2013–2014), Uganda (MIS 2014–2015) and Kenya (MIS 2015), where information on infection status using rapid diagnostic tests and/or light microscopy was available for 13,573 children. True prevalence was estimated using a Bayesian model that accounted for the conditional dependence between the two diagnostic methods, and the uncertainty of their sensitivities and specificities obtained from expert opinion.

**Results:**

The estimated true malaria prevalence was 20% (95% uncertainty interval [UI] 17%–23%) in the Democratic Republic of the Congo, 22% (95% UI 9–32%) in Uganda and 1% (95% UI 0–3%) in Kenya. According to the model estimations, rapid diagnostic tests had a satisfactory sensitivity and specificity, and light microscopy had a variable sensitivity, but a satisfactory specificity. Adding reported history of fever in the previous 14 days as a third diagnostic method to the model did not affect model estimates, highlighting the poor performance of this indicator as a malaria diagnostic.

**Conclusions:**

In the absence of a gold standard test, Bayesian models can assist in the optimal estimation of the malaria burden, using individual results from several tests and expert opinion about the performance of those tests.

**Electronic supplementary material:**

The online version of this article (10.1186/s12936-018-2211-y) contains supplementary material, which is available to authorized users.

## Background

Despite a worldwide decline in the malaria burden over the past 15 years, millions of people mainly across Africa still lack access to the tools they need to prevent and treat the disease. In 2015, about 200 million cases and 400,000 deaths due to malaria were estimated in the African continent, representing 90% of the world’s malaria burden [1].

In countries with poor systems for tracking malaria cases and deaths, as in sub-Saharan Africa countries, the prevalence of infections with malarial parasites is the most useful indicator of the level of transmission and, therefore, the most reliable means to understand the impact of control [2, 3]. Two diagnostic methods are commonly used in representative African household surveys to confirm whether an individual is infected or not with the malaria parasite, allowing for the estimation of the malaria prevalence [4, 5]. Light microscopy remains the reference method for laboratory confirmation of malaria, but it requires skilled microscopists and may be hampered by a poor quality of slide preparation or bad storage conditions [6, 7]. Rapid diagnostic tests offer a simple, fairly inexpensive, and reliable means of diagnosis that can be performed by health workers with limited training [8]; however, there are many available tests in the market with varying accuracy for the detection of malaria antigens or live parasite infections [9, 10]. In addition, household surveys collect information of history of fever in the previous 14 days, allowing for the estimation of the prevalence of febrile illness [4, 11].

In general, the sensitivity and specificity of a malaria diagnostic method may vary according to the characteristics of the surveyed population and other factors, such as age, sampling season, presence of cross-reacting diseases, malaria species, parasite density, and the quality of laboratory and experience of readers [9, 12]. As a result, the apparent prevalence estimated through these diagnostic tests may vary widely from one test to another and from one context to another. Indeed, the prevalence of malaria shown in various surveys differs according to the diagnostic method used [13–15].

The aim of this study was to estimate the true prevalence of malaria in children under five in the Democratic Republic of the Congo, Uganda and Kenya, as well as the sensitivity and specificity of the applied diagnostic methods. The true prevalence, based on the actual number of individuals infected by malaria parasite, instead of the apparent prevalence, based on the number of positive diagnostic methods, can be estimated in a Bayesian framework. This approach allows combining the opinion of experts on the diagnostic method characteristics with the obtained diagnostic method results into an optimal estimate of the “true” or “informed” prevalence [16–20].

## Methods

### Population and data collection

The Demographic and Health Surveys (DHS) and Malaria Indicator Surveys (MIS) are cross-sectional, nationally representative studies conducted in low and middle-income countries. For the current study, a DHS survey conducted in the Democratic Republic of the Congo from August 2013 to February 2014 was used [13], as well as two MIS surveys, one conducted in Uganda from December 2014 to January 2015 [14], and one in Kenya from July to August 2015 [15]. Malaria in children under five was diagnosed based on reported history of fever in the previous 14 days, rapid diagnostic tests and light microscopy.

The surveys used stratified multi-stage sampling. First, the countries were divided into small geographic areas (clusters) and in each cluster, three strata were created: towns, cities and rural or urban areas. In a second stage, households were randomly selected within each stratum. The interviews were conducted by health workers and/or technicians who received prior training on interviewing techniques, anthropometric measurement techniques, blood sampling, the performance of rapid diagnostic tests for the detection of malaria parasite antigens, and the preparation of thick films for the detection of parasites in blood.

During the survey, all children under 5 years old were identified, and their mother and/or guardian was asked whether or not the children had been ill with fever any time in the past 2 weeks. Then, a finger-prick blood sample was taken from each child for malaria diagnosis using light microscopy and rapid diagnostic tests (*SD Bioline malaria Ag*-*Pf* in the Democratic Republic of the Congo, *Paracheck*-*Pf* in Uganda, *SD Bioline malaria Ag*-*Pf/Pan* in Kenya). Thick and thin blood smears were prepared, dried, fixed with methanol, and stained with 2% Giemsa (a mixture of methylene blue, eosin, azure B and a solvent). Smears were periodically sent to a national reference laboratory for microscopic examination. Light microscopy was considered negative when the examination of 100 high power fields did not reveal asexual parasites or gametocytes [13–15].

### True prevalence estimation

The apparent malaria prevalence with corresponding 95% exact confidence intervals (CI) were primarily calculated for each country based on two test results: the outcomes of rapid diagnostic tests and light microscopy (i.e., positive or negative for malaria). Since the two test results cannot be considered completely independent of each other, a Bayesian framework to estimate the true malaria prevalence was developed to account for the conditional dependence between tests, and to incorporate external information on the sensitivity and specificity of those tests [20]. This modelling framework took advantage of previous work published by Branscum et al. [21] who modelled conditional dependence between two diagnostic methods. Both the design and sample weights of surveys were considered in the calculation of apparent and true prevalence [22].

The approach proposed by Branscum et al. [21] for two diagnostic methods models the apparent prevalence ($AP$) in terms of the true prevalence $TP$, the conditionally independent test sensitivities ($SE_{1} ,SE_{2}$) and the test specificities ($SP_{1} ,SP_{2}$), and the covariances between the two tests for infected and non-infected individuals ($a,b$):

$$AP_{11} = TP\left[ {SE_{1} SE_{2} + a} \right] + (1 - TP)\left[ {\left( {1 - SP_{1} } \right)\left( {1 - SP_{2} } \right) + b} \right]$$

$$AP_{10} = TP\left[ {SE_{1} (1 - SE_{2} ) - a} \right] + (1 - TP)\left[ {\left( {1 - SP_{1} } \right)SP_{2} - b} \right]$$

$$AP_{01} = TP\left[ {(1 - SE_{1} )SE_{2} - a} \right] + (1 - TP)\left[ {SP_{1} \left( {1 - SP_{2} } \right) - b} \right]$$

$$AP_{00} = TP\left[ {\left( {1 - SE_{1} } \right)(1 - SE_{2} ) + a} \right] + (1 - TP)\left[ {SP_{1} SP_{2} + b} \right]$$

where $AP_{11}$ is the apparent prevalence of positive results in both tests, $AP_{10}$ is the apparent prevalence of a positive result in the first test and a negative in the second, and so on.

This model was applied in the current study to include the results of two conditionally dependent tests—i.e., rapid diagnostic test ($T1$) and light microscopy ($T2$). The apparent test results are given by a vector $\varvec{x} = (x_{11} ,x_{10} ,x_{01} ,x_{00} )$, with $x_{11}$ the number of individuals testing positive in both tests, $x_{10}$ the number of individuals testing positive in the first but negative in the second, and so on. The vector $\varvec{x}$ was assumed to be distributed according to a multinomial distribution:

$$\varvec{x} \sim {\text{multinomial}}(n, \varvec{AP})$$

With $n = \mathop \sum \nolimits x$ the total sample size and $\varvec{AP} = (AP_{11} ,AP_{10} ,AP_{01} ,AP_{00} )$ the apparent prevalence vector corresponding to each possible combination of test results.

Expert opinion allowed deriving prior distributions for the test sensitivities and specificities. A Beta (1, 1) prior was applied for $TP$, and Uniform (−0.25, 0.25) priors for the covariance between two tests. The boundaries of the latter distribution correspond to the natural limits of the covariance parameter. To assess the impact of the informative priors for the sensitivities and specificities of the diagnostic methods, one-way sensitivity analyses were performed in which each informative prior was one-at-a time replaced by an uninformative Beta (1, 1) prior.

Models were implemented independently for the three countries. For each model, two chains of 110,000 iterations were simulated, of which the first 10,000 were discarded as burn-in. No thinning was applied. The convergence of the models was assessed by calculating the multivariate potential scale reduction factor [23] and by assessing trace and density plots. Posterior distributions were summarized using the mean and a 95% uncertainty interval (UI) defined as the distribution’s 2.5th and 97.5th percentile. All models were implemented using the prevalence package for R 3.4.3 [24], which builds on JAGS to perform Markov Chain Monte Carlo simulations using the Gibbs sampler [25].

In a scenario analysis, the 2-test covariance model was extended to include the results of reported history of fever in the previous 14 days as a third diagnostic method. A full description of this 3-test covariance model is included in Additional file 1.

### Prior information from experts

Prior information on the sensitivity and specificity of the diagnostic methods was obtained by sending questionnaires to experts—i.e., people with knowledge and expertise regarding malaria in the concerned countries. Experts who published on malaria diagnostic methods between 2005 and 2015 were identified through a PubMed search using the search phrase “malaria” AND “diagnostic test” AND (“Congo” OR “Uganda” OR “Kenya”).

The questionnaire elicited the experts’ knowledge on the sensitivity and specificity of rapid diagnostic tests light microscopy, and reported history of fever in the previous 14 days, for diagnosing malaria in children under five in the concerned countries (see Additional file 2). To capture the uncertainty in the elicited opinions and to keep the burden as minimal as possible, experts were asked to provide a minimal and maximal possible value. For each test characteristic, the prior information provided by the different experts were combined into a single Beta distribution. First, 10,000 random samples were generated per expert, assuming a uniform distribution between the experts’ minimal and maximal value. These simulations were then combined into a single vector, implying that all experts were given an equal weight. Finally, a Beta distribution was fitted to this joint distribution using maximum likelihood, via the fitdist function in the fitdistrplus package for R 3.4.3. [26]. Additional file 3 provides example R code for this process.

## Results

Survey data from 13,573 children under 5 years old were used in the analysis, including: 6941 children from the Democratic Republic of the Congo, 4072 children from Uganda, 2560 children from Kenya. Table 1 presents apparent malaria prevalence by country and diagnostic test. Across all tests, Kenya showed a lower apparent prevalence than the Democratic Republic of the Congo and Uganda. The apparent prevalence found by light microscopy was systematically lower than the ones based on rapid diagnostic tests.

**Table 1:** Positive samples ($\varvec{x}$) and apparent prevalence ($\varvec{AP}$, %) with 95% exact confidence interval for malaria by country and diagnostic method

Diagnostic test	DRC (n = 6941)		Uganda (n = 4072)		Kenya (n = 2560)	
	*x*	*AP (95% CI)*	*x*	*AP (95% CI)*	*x*	*AP (95% CI)*
RDT	2090	30 (29–31)	1210	30 (28–31)	209	8.2 (7.1–9.3)
Microscopy	1523	22 (21–23)	761	19 (18–20)	113	4.4 (3.7–5.3)

*RDT* rapid diagnostic test

Table 2 shows the obtained test results according to possible test combinations. The proportion of children who had a positive result with both tests was similar in the Democratic Republic of the Congo and Uganda, i.e., 18% (95% CI 17–19%) and 16% (95% CI 15–17%), respectively. In Kenya, only 3.7% (95% CI 3.0–4.5%) were positive on both tests. the proportion of children who had negative results on both tests was 66% (95% CI 65–67%) in the Democratic Republic of the Congo, 68% (95% CI 66–69%) in Uganda, and 91% (95% CI 90–92%) in Kenya.

**Table 2:** Number of individuals as a function of the results of the two diagnostic methods for the Democratic Republic of the Congo (n = 6941), Uganda (n = 4072) and Kenya (n = 2560)

Diagnostic test		Number of individuals, DRC	Number of individuals, Uganda	Number of individuals, Kenya
RDT	Microscopy			
1	1	1237	663	94
1	0	853	547	116
0	1	286	98	19
0	0	4565	2764	2332

*RDT* rapid diagnostic test

Prior information on the sensitivity and specificity of the diagnostic methods was available from six experts on malaria in sub-Saharan countries (one from the Democratic Republic of the Congo, two from Uganda and three from Kenya; see Additional file 4). Table 3 shows the resulting Beta distributions for the sensitivity and specificity of rapid diagnostic test and light microscopy in each considered country.

**Table 3:** Prior information on sensitivity and specificity of different diagnostic methods for malaria in three sub-Saharan countries

Diagnostic test	Sensitivity		Specificity	
	Fitted distribution	Mean (P025–P975)	Fitted distribution	Mean (P025–P975)
Democratic Republic of the Congo				
RDT	Beta (501, 44)	0.92 (0.90–0.94)	Beta (466, 67)	0.88 (0.85–0.90)
Microscopy	Beta (33, 1.7)	0.95 (0.86–1.00)	Beta (27, 3.3)	0.89 (0.76–0.97)
Uganda				
RDT	Beta (25, 4.5)	0.85 (0.70–0.95)	Beta (8.3, 2.5)	0.77 (0.49–0.96)
Microscopy	Beta (7.8, 5.7)	0.58 (0.32–0.82)	Beta (32, 1.7)	0.95 (0.86–1.00)
Kenya				
RDT	Beta (25, 6.2)	0.80 (0.65–0.92)	Beta (17, 7.8)	0.68 (0.49–0.85)
Microscopy	Beta (27, 7.5)	0.78 (0.64–0.90)	Beta (16, 3.5)	0.82 (0.62–0.95)

*RDT* rapid diagnostic test, *P025* 2.5th percentile, *P975* 97.5th percentile

Convergence was obtained for all three countries. Table 4 shows the estimated true malaria prevalence in the three considered countries, as well as the estimated diagnostic method characteristics. Similar true malaria prevalence was estimated for the Democratic Republic of the Congo (20%) and Uganda (22%), while the estimate for Kenya was considerably lower (1%). Across all countries, rapid diagnostic tests showed satisfactory sensitivities and specificities, while light microscopy had variable sensitivities, but satisfactory specificities. More detailed results, including convergence statistics for each parameter and Bayesian measures of complexity and fit, are available in Additional file 5. The one-way sensitivity analyses showed that all models were robust against the specification of each individual informative prior (see Additional file 6).

**Table 4:** Estimated (mean and 95% uncertainty interval) true malaria prevalence and diagnostic methods’ sensitivity and specificity by country

Parameter	DRC	Uganda	Kenya
True prevalence	0.20 (0.17–0.23)	0.22 (0.09–0.32)	0.01 (0.00–0.03)
RDT, sensitivity	0.92 (0.90–0.94)	0.84 (0.69–0.94)	0.78 (0.63–0.90)
RDT, specificity	0.86 (0.83–0.88)	0.86 (0.76–0.95)	0.92 (0.91–0.94)
Microscopy, sensitivity	0.90 (0.78–0.98)	0.61 (0.41–0.81)	0.77 (0.63–0.89)
Microscopy, specificity	0.95 (0.92–0.97)	0.93 (0.85–0.98)	0.96 (0.95–0.98)

*RDT* rapid diagnostic test, *DRC* the Democratic Republic of the Congo

Including reported history of fever in the previous 14 days as a third diagnostic method in the model did not influence the main model estimates (see Additional file 1). The estimated true prevalence remained nearly identical—i.e., 21% (95% UI 17–24%) in the Democratic Republic of the Congo, 22% (95% UI 8.9–33%) in Uganda and 1% (95% UI 0–3%) in Kenya. Across countries, the estimated sensitivity of reported history of fever in the previous 14 days as diagnostic method for malaria ranged from 53 to 57%, while its specificity ranged from 61 to 74%.

## Discussion

A Bayesian framework was used to estimate the true malaria prevalence from malaria household surveys in three sub-Saharan African countries, using survey results of two conditionally dependent diagnostic methods, and prior information on the conditionally independent sensitivities and specificities of evaluated tests [21]. The adopted modelling approach produced optimized prevalence estimations that allow comparability across different settings. Indeed, despite unprecedented levels of intervention coverage across sub-Saharan Africa since early 2000s [1], true malaria prevalence in children under five in two of the three selected countries (i.e., the Democratic Republic of the Congo and Uganda) was still estimated to be around 20%. In Kenya, on the other hand, the true malaria prevalence was estimated at 1% (even though the apparent prevalence varied from 4 to 40% according to the test, only 2% of children had a positive result for both tests).

Differences in the accuracy between diagnostic methods can result in a considerable variation in malaria prevalence estimations, when test sensitivity and specificity are not taken into account [19]. As found in the current study, the apparent malaria prevalence in each country varied widely according to the test used. In Uganda, for instance, the screening with rapid diagnostic tests yielded a malaria prevalence of 30%, while the microscopic detection of parasites in blood smears generated a much lower malaria prevalence of 19%. A similar observation was made in Kenya, where the malaria prevalence obtained with rapid diagnostic test was twice as high as that obtained with microscopic detection, i.e., 8% versus 4%. These differences are linked to a differential bias across diagnostic methods (e.g., more sub-microscopic cases in settings with lower transmission or higher levels of prior treatment [5]), resulting in different but unknown rates of false positive and false negative results. Indeed, in absence of a gold standard test [27], neither the sensitivity nor the specificity of the tests used in household surveys is known with certainty, introducing additional uncertainty (through Beta distributions of sensitivity and specificity values reported by malaria experts) when modelling apparent prevalence to estimate true prevalence.

Across countries, rapid diagnostic tests showed satisfactory sensitivities and specificities, while light microscopy had variable sensitivities, but satisfactory specificities. Indeed the estimated sensitivity of microscopy varied from 61% in Uganda over 77% in Kenya to 90% in the Democratic Republic of the Congo. In addition to differences in the observed numbers of positive and negative cases, this variability may also be a result of differences in expert opinion. Indeed, experts evaluated the microscopy results as showing more false negatives in Uganda compared to Kenya and the Democratic Republic of the Congo, with mean sensitivities of 58, 78 and 95%, respectively, which could be an indication of their differential evaluation of the environment (e.g., better microscopists, better storage conditions). In this respect, it can be noted that the values provided by the experts from Uganda were in line with some population-based survey studies that calculated the sensitivity of the microscopy using the PCR as gold standard (e.g., [5]). On the other hand, the values provided by the experts from Kenya and the Democratic Republic of the Congo seemed to be higher than those reported in population-based survey studies where PCR was used as gold standard (e.g., [28, 29]). Nonetheless, the one-way sensitivity analyses showed that the true prevalence estimates in all three countries were robust against misspecification of this prior.

Besides considering that none of diagnostic methods used in household surveys is the gold standard for malaria (imperfect tests) and that those diagnostic methods are not completely independent of each other, the adopted Bayesian modelling approach was able to impose consistency between the estimates for true prevalence and for the test characteristics. This allowed resolving possible discrepancies between the prior information (i.e., expert opinion about sensitivity and specificity) and the observed data, which are not unusual in Bayesian models [16]. For example, the low number of individuals with positive tests in Kenya was inconsistent with the moderate specificities elicited for rapid diagnostic test (68%) and light microscopy (82%), however specificities estimated using the Bayesian model were high (92 and 96%, respectively). On the other hand, it should be noted that Bayesian models with different dependency structures can provide different parameter estimates, in spite of their similarity in terms of adjustment measures. However, when only few tests are available, as in the current study, it may be nearly impossible to distinguish between different classes of models for the dependence structure, and unverifiable modelling assumptions are needed to make inference [30].

Extending the Branscum et al. [21] model to allow for simultaneous consideration of three conditionally dependent diagnostics methods, allowed evaluating the added value of reported fever history within the past 14 days as a malaria diagnostic. Although fever is used in the field as a proxy indicator of malaria illness, the added value of the DHS/MIS fever indicator in studying malaria epidemiology could not be shown. This is not unsurprising, as reported history of fever in the previous 14 days is too unspecific for current malaria status [31].

Accurate and timely information on numbers and trends in malaria-related cases and deaths is a key requirement for tracking the progress of malaria control and elimination efforts [32]. In countries with poor surveillance systems, such as in sub-Saharan Africa countries, this information can only be obtained by modelling the relationship between parasite prevalence and case incidence, or mortality [3, 33, 34]. The estimates intend to fill gaps in reported data [35]; but because they rely on relationships between variables that are uncertain, and draw on data that may be imprecisely measured, the estimates usually have a considerable degree of uncertainty [36]. Researchers from the Malaria Atlas Project (MAP) have developed a Bayesian hierarchical model intended to handle the different sources of uncertainty (e.g., uncertainty in the sample size and spatio-temporal density of prevalence surveys, uncertainty in the relationship between malaria prevalence and environmental covariates) with the aim of predicting a spatio-temporal cube of *Plasmodium falciparum* prevalence at 5 × 5 km resolution across all endemic African countries [3, 33]. However, most malaria risk maps produced by this global initiative [3, 33] and by other research groups [37] did not consider that malaria prevalence from national and sub-national household surveys can be influenced by the uncertainty in the accuracy of diagnostic methods used during the surveys. Repeating the here adopted approach across multiple countries and with more experts may, therefore, provide a potential contribution to the current MAP results.

The main limitations of the current study may be related to the assumption of a single sensitivity and specificity for each diagnostic method within each country, irrespective of the endemicity and dynamics of the malaria transmission at sub-national and local levels. A negative correlation between the intensity of malaria transmission and the proportion of infections with low parasite densities has been repeatedly described [38, 39], thus influencing the limit of detection of light microscopy and rapid diagnostic tests and consequently the sensitivity of those tests. The geographical variation in the presence of histidine-rich protein 2 (Pfhrp2) in *P. falciparum* parasites across the country can also influence the accuracy of rapid diagnostic tests for detecting malaria [40]. This spatial heterogeneity in the performance of diagnostic methods can be taken into account in the approach presented here, but it will require more detailed information from malaria experts about the relationships between the performance of diagnostic methods and relevant covariates, as well as information on the distribution of these covariates [20].

## Conclusion

In absence of a true gold standard test, Bayesian modelling remains a powerful tool for estimating true malaria burden. The combination of results from two malaria diagnostic methods used in household surveys with expert opinion about the performance of the diagnostic methods offers the possibility to compare prevalence estimates between countries and over time in a same country. This comparability is key when monitoring and evaluating malaria control and elimination efforts.

## Additional files

**Additional file 1.** Scenario analysis including fever, rapid diagnostic test, and light microscopy.

**Additional file 2.** Expert opinion questionnaire.

**Additional file 3.** Example R code to fit Beta distribution to expert opinion.

**Additional file 4.** Results of the expert opinion survey.

**Additional file 5.** Detailed output of the 2-test covariance model.

**Additional file 6.** One-way sensitivity analyses.
