# Supplementary material for: True malaria prevalence in children under five: Bayesian estimation using data of malaria household surveys from three sub-Saharan countries
Source: Malar J. 2018 Feb 5;17:65. doi: 10.1186/s12936-018-2211-y (PMC5800038; doi:10.1186/s12936-018-2211-y)
Supplement: Supplementary file 1 — Additional file 1. Scenario analysis including fever, rapid diagnostic test, and light microscopy. [file 12936_2018_2211_MOESM1_ESM.docx]

True malaria prevalence in children under five: Bayesian estimation using data of malaria household surveys from three sub-Saharan countries

# Additional file 1. Scenario analysis including fever, rapid diagnostic test, and light microscopy

## Methods

The 2-test covariance model proposed by Branscum et al. [1] was extended in a scenario analysis to include the results of three conditionally dependent tests – i.e., reported history of fever in the previous 14 days ($T1$), rapid diagnostic test ($T2$) and light microscopy ($T3$). The apparent test results are now given by a vector $\boldsymbol{x}=(x_{111},x_{110},x_{101},x_{100},x_{011},x_{010},x_{001},x_{000})$, with $x_{111}$the number of individuals testing positive in all three tests, $x_{110}$ the number of individuals testing positive in the first and second test but negative in the third, and so on. The vector $\boldsymbol{x}$ was assumed to be distributed according to a multinomial distribution:

$$\boldsymbol{x} \sim multinomial(n, \boldsymbol{AP})$$

With $n=\sum x$the total sample size and $\boldsymbol{AP}=\left( {AP}_{111},{AP}_{110},{AP}_{101},{AP}_{100},{AP}_{011},{AP}_{010},{AP}_{001},{AP}_{000} \right)$the apparent prevalence vector corresponding to each possible combination of test results. According with the extended model, $\boldsymbol{AP}$ is defined in terms of the true prevalence ($TP$), the conditionally independent test sensitivities ($SE_{1},SE_{2},SE_{3}$) and test specificities ($SP_{1},SP_{2},SP_{3}$), the covariances between each pair of tests for infected individuals ($a_{1},a_{2},a_{3}$) and non-infected individuals ($b_{1},b_{2},b_{3}$), and the covariances between the three tests for infected and non-infected individuals ($a_{4},b_{4}$). The complete definition of $\boldsymbol{AP}$is given below.

A Beta (1, 1) prior was applied for $TP$, Uniform (-0.25, 0.25) priors for the covariance between two tests, and Uniform (-0.125, 0.125) priors for the covariance between three tests. The boundaries of the latter two distributions corresponded to the natural limits of the respective covariance parameters.

### Definition of apparent prevalence in the 3-test covariance model

$${AP}_{111}=TP\left[ SE_{1}SE_{2}SE_{3}+a_{1}SE_{3}+a_{2}SE_{2}+a_{3}SE_{1}+a_{4} \right]+\left( 1-TP \right)\left[ \left( 1-SP_{1} \right)\left( 1-SP_{2} \right)\left( 1-SP_{3} \right)+b_{1}\left( 1-SP_{3} \right)+b_{2}\left( 1-SP_{2} \right)+b_{3}\left( 1-SP_{1} \right)-b_{4} \right]$$

$${AP}_{110}=TP\left[ SE_{1}SE_{2}\left( 1-SE_{3} \right)+a_{1}\left( 1-SE_{3} \right)-a_{2}SE_{2}-a_{3}SE_{1}-a_{4} \right]+(1-TP)\left[ \left( 1-SP_{1} \right)\left( 1-SP_{2} \right)SP_{3}+b_{1}SP_{3}-b_{2}\left( 1-SP_{2} \right)-b_{3}\left( 1-SP_{1} \right)+b_{4} \right]$$

$${AP}_{101}=TP\left[ SE_{1}\left( 1-SE_{2} \right)SE_{3}-a_{1}SE_{3}+a_{2}\left( 1-SE_{2} \right)-a_{3}SE_{1}-a_{4} \right]+\left( 1-TP \right)\left[ \left( 1-SP_{1} \right)SP_{2}\left( 1-SP_{3} \right)-b_{1}\left( 1-SP_{3} \right)+b_{2}SP_{2}-b_{3}\left( 1-SP_{1} \right)+b_{4} \right]$$

$${AP}_{100}=TP\left[ SE_{1}\left( 1-SE_{2} \right)\left( 1-SE_{3} \right)-a_{1}\left( 1-SE_{3} \right)-a_{2}\left( 1-SE_{2} \right)+a_{3}SE_{1}+a_{4} \right]+(1-TP)\left[ \left( 1-SP_{1} \right)SP_{2}SP_{3}-b_{1}SP_{3}-b_{2}SP_{2}+b_{3}\left( 1-SP_{1} \right)-b_{4} \right]$$

$${AP}_{011}=TP\left[ \left( 1-SE_{1} \right)SE_{2}SE_{3}-a_{1}SE_{3}-a_{2}SE_{2}+a_{3}\left( 1-SE_{1} \right)-a_{4} \right]+\left( 1-TP \right)\left[ SP_{1}\left( 1-SP_{2} \right)\left( 1-SP_{3} \right)-b_{1}\left( 1-SP_{3} \right)-b_{2}\left( 1-SP_{2} \right)+b_{3}SP_{1}+b_{4} \right]$$

$${AP}_{010}=TP\left[ \left( 1-SE_{1} \right)SE_{2}\left( 1-SE_{3} \right)-a_{1}\left( 1-SE_{3} \right)+a_{2}SE_{2}-a_{3}(1-SE_{1})+a_{4} \right]+(1-TP)\left[ SP_{1}\left( 1-SP_{2} \right)SP_{3}-b_{1}SP_{3}+b_{2}\left( 1-SP_{2} \right)-b_{3}SP_{1}-b_{4} \right]$$

$${AP}_{001}=TP\left[ \left( 1-SE_{1} \right)\left( 1-SE_{2} \right)SE_{3}+a_{1}SE_{3}-a_{2}\left( 1-SE_{2} \right)-a_{3}(1-SE_{1})+a_{4} \right]+\left( 1-TP \right)\left[ SP_{1}SP_{2}\left( 1-SP_{3} \right)+b_{1}\left( 1-SP_{3} \right)-b_{2}SP_{2}-b_{3}SP_{1}-b_{4} \right]$$

$${AP}_{000}=TP\left[ \left( 1-SE_{1} \right)\left( 1-SE_{2} \right)\left( 1-SE_{3} \right)+a_{1}\left( 1-SE_{3} \right)+a_{2}\left( 1-SE_{2} \right)+a_{3}\left( 1-SE_{1} \right)-a_{4} \right]+(1-TP)\left[ SP_{1}SP_{2}SP_{3}+b_{1}SP_{3}+b_{2}SP_{2}+b_{3}SP_{1}+b_{4} \right]$$

## Results

**Table A1. Positive samples (**$\boldsymbol{x}$**) and apparent prevalence (**$\boldsymbol{AP}$**, %) with 95% exact confidence interval for malaria by country and diagnostic test.**

| **Diagnostic test** | **DRC (n = 6** **941)** | | **Uganda (n = 4** **072)** | | **Kenya (n = 2** **560)** | |
| --- | --- | --- | --- | --- | --- | --- |
|  | *x* | *AP (95%CI)* | *x* | *AP (95%CI)* | *x* | *AP (95%CI)* |
| Fever | 2184 | 31 (30–33) | 1342 | 33 (32–34) | 1007 | 39 (37–41) |
| RDT | 2090 | 30 (29–31) | 1210 | 30 (28–31) | 209 | 8.2 (7.1–9.3) |
| Microscopy | 1523 | 22 (21–23) | 761 | 19 (18–20) | 113 | 4.4 (3.7–5.3) |

*Fever = Reported fever history in the previous 14 days; RDT = Rapid Diagnostic Test*

**Table A2. Number of individuals as a function of the results of the three diagnostic methods for the Democratic Republic of the Congo (n = 6** **941), Uganda (n = 4** **072) and Kenya (n = 2** **560).**

| **Diagnostic test** | | | **Number of individuals, DRC** | **Number of individuals, Uganda** | **Number of individuals, Kenya** |
| --- | --- | --- | --- | --- | --- |
| *Fever* | *RDT* | *Microscopy* |  |  |  |
| 1 | 1 | 1 | 490 | 285 | 54 |
| 1 | 1 | 0 | 365 | 329 | 85 |
| 1 | 0 | 1 | 98 | 38 | 12 |
| 1 | 0 | 0 | 1 231 | 690 | 857 |
| 0 | 1 | 1 | 747 | 378 | 40 |
| 0 | 1 | 0 | 488 | 218 | 31 |
| 0 | 0 | 1 | 188 | 60 | 7 |
| 0 | 0 | 0 | 3 334 | 2 074 | 1 475 |

*Fever = Reported fever history in the previous 14 days; RDT = Rapid Diagnostic Test*

**Table A3. Prior information on sensitivity and specificity of different diagnostic methods for malaria in three sub-Saharan countries.**

| **Diagnostic test** | **Sensitivity** | | **Specificity** | |
| --- | --- | --- | --- | --- |
|  | *Fitted distribution* | *Mean (P025-P975)* | *Fitted distribution* | *Mean (P025-P975)* |
| *Democratic Republic of the Congo* | | | | |
| Fever | Beta (16,1.7) | 0.90 (0.73–0.99) | Beta (48,6.9) | 0.88 (0.78–0.95) |
| RDT | Beta (501,44) | 0.92 (0.90–0.94) | Beta (466,67) | 0.88 (0.85–0.90) |
| Microscopy | Beta (33,1.7) | 0.95 (0.86–1.00) | Beta (27,3.3) | 0.89 (0.76–0.97) |
| *Uganda* | | | | |
| Fever | Beta (2.3,2.4) | 0.49 (0.11–0.88) | Beta (48,8.4) | 0.85 (0.75–0.93) |
| RDT | Beta (25,4.5) | 0.85 (0.70–0.95) | Beta (8.3,2.5) | 0.77 (0.49–0.96) |
| Microscopy | Beta (7.8,5.7) | 0.58 (0.32–0.82) | Beta (32,1.7) | 0.95 (0.86–1.00) |
| *Kenya* | | | | |
| Fever | Beta (3.2,2.2) | 0.59 (0.20–0.92) | Beta (18,25) | 0.42 (0.27–0.57) |
| RDT | Beta (25,6.2) | 0.80 (0.65–0.92) | Beta (17, 7.8) | 0.68 (0.49–0.85) |
| Microscopy | Beta (27,7.5) | 0.78 (0.64–0.90) | Beta (16,3.5) | 0.82 (0.62–0.95) |

*RDT = Rapid Diagnostic Test; P025: 2.5^th^ percentile; P975: 97.5^th^ percentile*

**Table A4. Estimated (mean and 95% uncertainty interval) true malaria prevalence and diagnostic methods’ sensitivity and specificity by country.**

| **Parameter** | **DRC** | **Uganda** | **Kenya** |
| --- | --- | --- | --- |
| True prevalence | 0.21 (0.17–0.24) | 0.22 (0.09–0.33) | 0.01 (0.00–0.03) |
| Fever, sensitivity | 0.53 (0.43–0.65) | 0.57 (0.37–0.77) | 0.55 (0.24–0.89) |
| Fever, specificity | 0.74 (0.71–0.77) | 0.74 (0.68–0.81) | 0.61 (0.59–0.62) |
| RDT, sensitivity | 0.92 (0.89–0.94) | 0.80 (0.66–0.91) | 0.76 (0.61–0.88) |
| RDT, specificity | 0.86 (0.83–0.89) | 0.84 (0.75–0.93) | 0.92 (0.91–0.94) |
| Microscopy, sensitivity | 0.78 (0.68–0.91) | 0.55 (0.38–0.74) | 0.74 (0.60–0.86) |
| Microscopy, specificity | 0.93 (0.89–0.96) | 0.91 (0.85–0.97) | 0.96 (0.95–0.97) |

*Fever = Reported fever history in the previous 14 days; RDT = Rapid Diagnostic Test; DRC = the Democratic Republic of the Congo*

## References

1. Branscum AJ, Gardner IA, Johnson WO. Bayesian modeling of animal- and herd-level prevalences. Prev. Vet. Med. 2004;66:101–12.
