# Supplementary material for: True malaria prevalence in children under five: Bayesian estimation using data of malaria household surveys from three sub-Saharan countries
Source: Malar J. 2018 Feb 5;17:65. doi: 10.1186/s12936-018-2211-y (PMC5800038; doi:10.1186/s12936-018-2211-y)
Supplement: Supplementary file 2 — Additional file 2. Expert opinion questionnaire. [file 12936_2018_2211_MOESM2_ESM.docx]

True malaria prevalence in children under five: Bayesian estimation using data of malaria household surveys from three sub-Saharan countries

# Additional file 2. Expert opinion questionnaire

**Study on the true prevalence of malaria among children under five**

Malaria is a preventable and treatable illness but the lack of good quality data limits the design and implementation of preventive measures. The eventual eradication of malaria as a WHO goal requires a rigorous assessment of the malaria prevalence. Even if diagnostic tests are conducted during national health surveys such as the Demographic and Health Survey (DHS), sensitivity and specificity of a diagnostic test may vary according the characteristics of surveyed population and external factors (age, sampling season, presence of cross-reacting diseases, the quality of laboratory and the experience of the readers in the case of microscopy, etc).

The aim of this study is to estimate the true prevalence (based on the actual number of sick people, different to the apparent prevalence that takes into account the number of positive tests estimated by a diagnostic test) of malaria for under-five children.

Bayesian statistics are helpful to estimate the true prevalence without the need of a gold standard and the tests’ characteristics. This approach combines the prior information (from experts’ opinion) to the information provided by the data (malaria prevalence calculated with DHS data using three diagnostics tests: RDT: rapid diagnostic test, fever, microscopy: thick blood smears) to obtain the estimation of the malaria true prevalence. The true prevalence is calculated with a set of equations including the apparent prevalence (given by the data) and the tests characteristics (specificity and sensitivity, given by the experts) ^[[1]](#footnote-1)^. These equations use conditional probabilities e.g. probability of test 2 to be positive if the test 1 is positive knowing that test 1 is more sensitive than the test 2. The compromise between the survey results and data from experts then lead to an optimal estimate of the true prevalence.

## Background for Congo survey

The survey was conducted from August 2013 to February 2014, during dry and rainy seasons. Health workers (83 supervisors and 626 interviewers) were trained for several weeks on interview techniques; filling the questionnaires; anthropometric measurement techniques, sampling of blood, realization of Rapid Diagnostic Test (RDT) for malaria and blood collection on blades for thick film.

During the survey, interviewed women with children aged 0-59 months were asked about fever in their children (i.e., has your child been ill with fever any time in last two weeks?).

Malaria parasitemia was measured in two ways. In the field, health technicians used RDT (SD Bioline Malaria Ag Pf) to determine whether children had malaria infection from a finger-prick blood sample and from the same finger-prick thick and thin blood smears were prepared. The blood smears were dried and packed carefully in the field (fixed with methanol as soon as the smear is dry). Thick and thin blood smears were stained with 2 percent Giemsa (a mixture of methylene blue, eosin, azure B and a solvent). They were periodically sent to National Reference Laboratory of the National Program for the Fight against sexually transmitted infections in Kinshasa for microscopic examination in collaboration with the Parasitology laboratory in Medicine Faculty of the University of Kinshasa. A thick blood smear was considered negative when the examination of times 100 high power fields did not reveal asexual parasites or gametocytes. Results of the two laboratories were identical in 99.4% of cases, which confirms the good quality of work.

**Please take into account the following in answering the questions:**

1. Please refer to the context described above

2. In the DHS surveys, "fever" is taken as defined by a mother saying her child has had a fever in the last two weeks. So, the "fever" prevalence in our study depends on the community meaning of fever.

3. Microscopy in national survey may be of low quality (influencing the test reliability and validity) because of poor staining, preparation and storage of blood samples.

## Background for Kenya survey

The survey in Kenya was conducted from July to September 2010, during the dry season. Health workers (28 clinicians and 28 laboratory technologists) were trained on how to conduct informed consent and specimen collection procedures like preparing blood smears and performing rapid diagnostic tests (RDT) for malaria testing. Participants also received refresher training on the management of uncomplicated malaria and referral of complicated malaria cases.

During the survey, interviewed women with children aged 0-59 months were asked about fever in their children (i.e., has your child been ill with fever any time in last two weeks?).

Malaria parasitemia was measured in two ways. In the field, health technicians used RDT (CareStart Malaria HRP2/pLDH) to determine whether children had malaria infection from a finger-prick blood sample and from the same finger-prick thick and thin blood smears were prepared. The blood smears were dried and packed carefully in the field (fixed with methanol as soon as the smear is dry). Thick and thin blood smears were stained with 2 percent Giemsa (a mixture of methylene blue, eosin, azure B and a solvent) for 30 minutes. They were periodically sent by courier to the KEMRI/Walter Reed Project Malaria Diagnostics Centre laboratory in Kisumu for microscopic examination. A thick blood smear was considered negative when the examination of times 100 high power fields did not reveal asexual parasites or gametocytes. The protocol for the blood specimen collection and analysis was approved by the Kenyatta National Hospital/ University of Nairobi Scientific and Ethics Review Committee and ICF Macro’s Institutional Review Board.

**Please take into account the following in answering the questions:**

1. Please refer to the context described above

2. In the DHS surveys, "fever" is taken as defined by a mother saying her child has had a fever in the last two weeks. So, the "fever" prevalence in our study depends on the community meaning of fever.

3. Microscopy in national survey may be of low quality (influencing the test reliability and validity) because of poor staining, preparation and storage of blood samples.

## Background for Uganda survey

The malaria indicators survey in Uganda was conducted from November to December 2009. It was implemented by the Uganda Bureau of Statistics (UBOS) and the Uganda Malaria Surveillance Project (UMSP) on behalf of the National Malaria Control Program (NMCP). Health workers (59 interviewers, 18 nurses and 39 laboratory technicians) were trained on how to conduct informed consent and specimen collection procedures like preparing blood smears and performing rapid diagnostic tests (RDT) for malaria testing.

During the survey, interviewed women with children aged 0-59 months were asked about fever in their children (i.e., has your child been ill with fever any time in last two weeks?).

Malaria parasitemia was measured in two ways. In the field, health technicians used RDT (Paracheck Pf) to determine whether children had malaria infection from a finger-prick blood sample and from the same finger-prick thick and thin blood smears were prepared. The blood smears were dried and packed carefully in the field (fixed with methanol as soon as the smear is dry). They were periodically sent to the Molecular Laboratory at the Mulago Hospital in Kampala for microscopic examination**.** Thick and thin blood smears were stained with 2 percent Giemsa (a mixture of methylene blue, eosin, azure B and a solvent) for 30 minutes. A thick blood smear was considered negative when the examination of times 100 high power fields did not reveal asexual parasites or gametocytes. For quality control, all slides were read by a second microscopist, and a third reviewer settled any discrepant readings. Of the 4,075 thick slides evaluated, there were only 176 (4.3 percent) discrepant readings that were settled by a third reviewer.

**Please take into account the following in answering the questions:**

1. Please refer to the context described above

2. In the DHS surveys, "fever" is taken as defined by a mother saying her child has had a fever in the last two weeks. So, the "fever" prevalence in our study depends on the community meaning of fever.

3. Microscopy in national survey may be of low quality (influencing the test reliability and validity) because of poor staining, preparation and storage of blood samples.

# Table A. Dependence between tests

Two tests are **independent** when the sensitivity (or specificity) of one test does not depend on whether results of the other test are positive or negative among infected (or non-infected) individuals. Dependence between tests may happen when tests measure similar biological processes. In this case, false negative (or false positive) results may be correlated. Two tests would be completely dependent if e.g. among infected individuals tested negative with one test, the other test would fail to detect additional infected individuals.

|  | **Microscopy and RDT** | **Microscopy and fever** | **Fever and RDT** |
| --- | --- | --- | --- |
| **For malaria infected children:** |  |  |  |
| Are both tests independent? Please answer with **YES** or **NO**. |  |  |  |
| If not, is the association between these two tests always **POSITIVE** *(e.g. if one test has a positive result, it is more likely that the other test will also be positive)* or always **NEGATIVE** *(e.g. if one test has a positive result, it is more likely that the other test will be negative)*, or could it be **BOTH** *(e.g., depending on the sample, the patient, etc.)*? |  |  |  |
| **For malaria non infected children:** |  |  |  |
| Are both tests independent? Please answer with **YES** or **NO**. |  |  |  |
| If not, is the association between these two tests always **POSITIVE** *(e.g. if one test has a negative result, it is more likely that the other test will also be negative)* or always **NEGATIVE** *(e.g. if one test has a negative result, it is more likely that the other test will be positive)*, or could it be **BOTH** *(e.g., depending on the sample, the patient, etc.)*? |  |  |  |

# Table B. Prior information

Please give an estimation of the expected **MINIMAL** and **MAXIMAL** probability for the following parameters; e.g. [60% – 100%] would correspond to the situation where the parameter is believed to be between 60% and 100%. MIN/MAX values may not be lower than 0% or higher than 100%. Optionally, you may provide comments to explain your responses. We have divided this table in two parts: in high transmission area and in low transmission area.

| **Parameter** | **MIN** | **MAX** | **Comments (optional)** |
| --- | --- | --- | --- |
| What is the **sensitivity** of the **microscopy test** for the detection of a child infected with malaria? In other words, what is the probability that a child **infected with malaria** will be positive with **microscopy test**? |  |  |  |
| What is the **specificity** of the **microscopy test** for the detection of a child not infected with malaria? In other words, what is the probability that a child **not infected with malaria** will be negative with **microscopy test**? |  |  |  |
| What is the **sensitivity** of the **RDT** for the detection of a child infected with malaria? In other words, what is the probability that a child **infected with malaria** will be **positive** with **RDT**? |  |  |  |
| What is the **specificity** of the **RDT** for the detection of a child not infected with malaria? In other words, what is the probability that a child **not infected with malaria** will test **negative** with **RDT**? |  |  |  |
| What is the **sensitivity** of the **fever** for the detection of a child infected with malaria? In other words, what is the probability that a child **infected with malaria** to have **fever**? |  |  |  |
| What is the **specificity** of **fever** for the detection of a child not infected with malaria? In other words, what is the probability that a child **not infected with malaria** to do not have **fever**? |  |  |  |

1. Niko Speybroeck and *al.* True versus Apparent Malaria Infection Prevalence: The Contribution of a Bayesian Approach. February 18, 2011DOI: 10.1371/journal.pone.0016705 [↑](#footnote-ref-1)
