# Supplementary material for: True malaria prevalence in children under five: Bayesian estimation using data of malaria household surveys from three sub-Saharan countries
Source: Malar J. 2018 Feb 5;17:65. doi: 10.1186/s12936-018-2211-y (PMC5800038; doi:10.1186/s12936-018-2211-y)
Supplement: Supplementary file 3 — Additional file 3. Example R code to fit Beta distribution to expert opinion. [file 12936_2018_2211_MOESM3_ESM.docx]

True malaria prevalence in children under five: Bayesian estimation using data of malaria household surveys from three sub-Saharan countries

# Additional file 3. Example R code to fit Beta distribution to expert opinion.

### FIT BETA DISTRIBUTION TO EXPERT OPINION

### UGANDA

## required packages

library(fitdistrplus)

## import expert opinion

df <-

structure(

list(SE_MIC = c(0.40, 0.80, 0.30, 0.80),

SP_MIC = c(0.90, 1.00, 0.90, 1.00),

SE_RDT = c(0.70, 0.95, 0.80, 0.95),

SP_RDT = c(0.65, 0.97, 0.50, 0.95),

SE_FEV = c(0.20, 0.80, 0.05, 0.90),

SP_FEV = c(0.75, 0.95, 0.80, 0.90)),

.Names = c("SE_MIC", "SP_MIC", "SE_RDT", "SP_RDT", "SE_FEV", "SP_FEV"),

row.names = c("MIN", "MAX", "MIN__1", "MAX__1"),

class = "data.frame")

## main wrapper function

fit <-

function(x, n = 1e4) {

if (!is.list(x)) x <- list(x)

sim <- unlist(lapply(x, function(x) runif(n, x[1], x[2])))

fit <- fitdist(sim, "beta")

out <- list(a = fit$estimate[1], b = fit$estimate[2])

class(out) <- "fit"

return(out)

}

## summary method for S3 class 'fit'

summary.fit <-

function(object, ...) {

beta <- sapply(object, formatC, format = "fg", digits = 2, width = 0)

beta <- paste0("Beta(", paste(beta, collapse = ","), ")")

stats <-

c(object$a / (object$a + object$b),

qbeta(c(0.025, 0.975), object$a, object$b))

names(stats) <- c("mean", "2.5%", "97.5%")

stats <- formatC(stats, format = "f", digits = 2)

stats <- paste0(stats[1], " (", paste(stats[2:3], collapse = "-"), ")")

return(list(beta, stats))

}

## fit Beta distributions to expert opinion

set.seed(264)

SE1 <- fit(list(df$SE_FEV[1:2], df$SE_FEV[3:4]))

SE2 <- fit(list(df$SE_RDT[1:2], df$SE_RDT[3:4]))

SE3 <- fit(list(df$SE_MIC[1:2], df$SE_MIC[3:4]))

SP1 <- fit(list(df$SP_FEV[1:2], df$SP_FEV[3:4]))

SP2 <- fit(list(df$SP_RDT[1:2], df$SP_RDT[3:4]))

SP3 <- fit(list(df$SP_MIC[1:2], df$SP_MIC[3:4]))

## summarize Beta distributions

SeSp <-

rbind(

summary(SE1), summary(SP1),

summary(SE2), summary(SP2),

summary(SE3), summary(SP3))

rownames(SeSp) <- c("SE1", "SP1", "SE2", "SP2", "SE3", "SP3")

colnames(SeSp) <- c("Distribution", "Mean (95%CI)")

print(SeSp)

## Distribution Mean (95%CI)

## SE1 "Beta(2.3,2.4)" "0.49 (0.11-0.88)"

## SP1 "Beta(48,8.4)" "0.85 (0.75-0.93)"

## SE2 "Beta(25,4.5)" "0.85 (0.70-0.95)"

## SP2 "Beta(8.3,2.5)" "0.77 (0.49-0.96)"

## SE3 "Beta(7.8,5.7)" "0.58 (0.32-0.82)"

## SP3 "Beta(32,1.7)" "0.95 (0.86-1.00)"
