# Supplementary material for: True malaria prevalence in children under five: Bayesian estimation using data of malaria household surveys from three sub-Saharan countries
Source: Malar J. 2018 Feb 5;17:65. doi: 10.1186/s12936-018-2211-y (PMC5800038; doi:10.1186/s12936-018-2211-y)
Supplement: Supplementary file 4 — Additional file 4. Results of the expert opinion survey. [file 12936_2018_2211_MOESM4_ESM.docx]

True malaria prevalence in children under five: Bayesian estimation using data of malaria household surveys from three sub-Saharan countries

# Additional file 4. Results of the expert opinion survey.

**Table D1. Expert opinion for diagnostic test sensitivity and specificity in Democratic Republic of the Congo.**

| **Parameter** | **Expert 1** | |
| --- | --- | --- |
|  | *Min* | *Max* |
| Fever, sensitivity | 0.80 | 1.00 |
| Fever, specificity | 0.80 | 0.95 |
| RDT, sensitivity | 0.90 | 0.94 |
| RDT, specificity | 0.85 | 0.90 |
| Microscopy, sensitivity | 0.90 | 1.00 |
| Microscopy, specificity | 0.80 | 0.98 |

*Fever = Reported fever history in the previous 14 days; RDT = Rapid Diagnostic Test*

**Table D2. Expert opinion for diagnostic test sensitivity and specificity in Uganda.**

| **Parameter** | **Expert 1** | | **Expert 2** | |
| --- | --- | --- | --- | --- |
|  | *Min* | *Max* | *Min* | *Max* |
| Fever, sensitivity | 0.20 | 0.80 | 0.05 | 0.90 |
| Fever, specificity | 0.75 | 0.95 | 0.80 | 0.90 |
| RDT, sensitivity | 0.70 | 0.95 | 0.80 | 0.95 |
| RDT, specificity | 0.65 | 0.97 | 0.50 | 0.95 |
| Microscopy, sensitivity | 0.40 | 0.80 | 0.30 | 0.80 |
| Microscopy, specificity | 0.90 | 1.00 | 0.90 | 1.00 |

*Fever = Reported fever history in the previous 14 days; RDT = Rapid Diagnostic Test*

**Table D3. Expert opinion for diagnostic test sensitivity and specificity in Kenya.**

| **Parameter** | **Expert 1** | | **Expert 2** | | **Expert 3** | |
| --- | --- | --- | --- | --- | --- | --- |
|  | *Min* | *Max* | *Min* | *Max* | *Min* | *Max* |
| Fever, sensitivity | 0.80 | 0.90 | 0.40 | 0.50 | 0.30 | 0.60 |
| Fever, specificity | 0.40 | 0.50 | 0.40 | 0.50 | 0.20 | 0.50 |
| RDT, sensitivity | 0.80 | 0.90 | 0.70 | 0.90 | 0.60 | 0.90 |
| RDT, specificity | 0.70 | 0.80 | 0.50 | 0.65 | 0.60 | 0.85 |
| Microscopy, sensitivity | 0.75 | 0.85 | 0.70 | 0.85 | 0.60 | 0.95 |
| Microscopy, specificity | 0.85 | 0.95 | 0.60 | 0.90 | 0.70 | 0.90 |

*Fever = Reported fever history in the previous 14 days; RDT = Rapid Diagnostic Test*
