# Supplementary material for: True malaria prevalence in children under five: Bayesian estimation using data of malaria household surveys from three sub-Saharan countries
Source: Malar J. 2018 Feb 5;17:65. doi: 10.1186/s12936-018-2211-y (PMC5800038; doi:10.1186/s12936-018-2211-y)

True malaria prevalence in children under five: Bayesian estimation using data of malaria household surveys from three sub-Saharan countries

# Additional file 5. Detailed output of the 2-test covariance model.

## Democratic Republic of the Congo

### Summary

## mean median mode sd 2.5% 97.5%

## TP 0.200 0.200 0.198 0.013 0.174 0.228

## SE[1] 0.920 0.921 0.921 0.011 0.897 0.941

## SE[2] 0.896 0.902 0.920 0.050 0.784 0.975

## SP[1] 0.855 0.854 0.852 0.012 0.832 0.880

## SP[2] 0.949 0.950 0.952 0.011 0.924 0.967

## a[1] 0.020 0.016 0.001 0.020 -0.009 0.064

## b[1] 0.005 0.003 -0.003 0.008 -0.006 0.025

##

## Multivariate BGR statistic = 1.0023

## BGR values substantially above 1 indicate lack of convergence

### Diagnostics

## $DIC

## Mean deviance: 52.31

## penalty 2.965

## Penalized deviance: 55.27

##

## $BGR

## Potential scale reduction factors:

##

## Point est. Upper C.I.

## SE[1] 1 1.00

## SE[2] 1 1.00

## SP[1] 1 1.00

## SP[2] 1 1.00

## TP 1 1.00

## a 1 1.01

## b 1 1.01

##

## Multivariate psrf

##

## 1


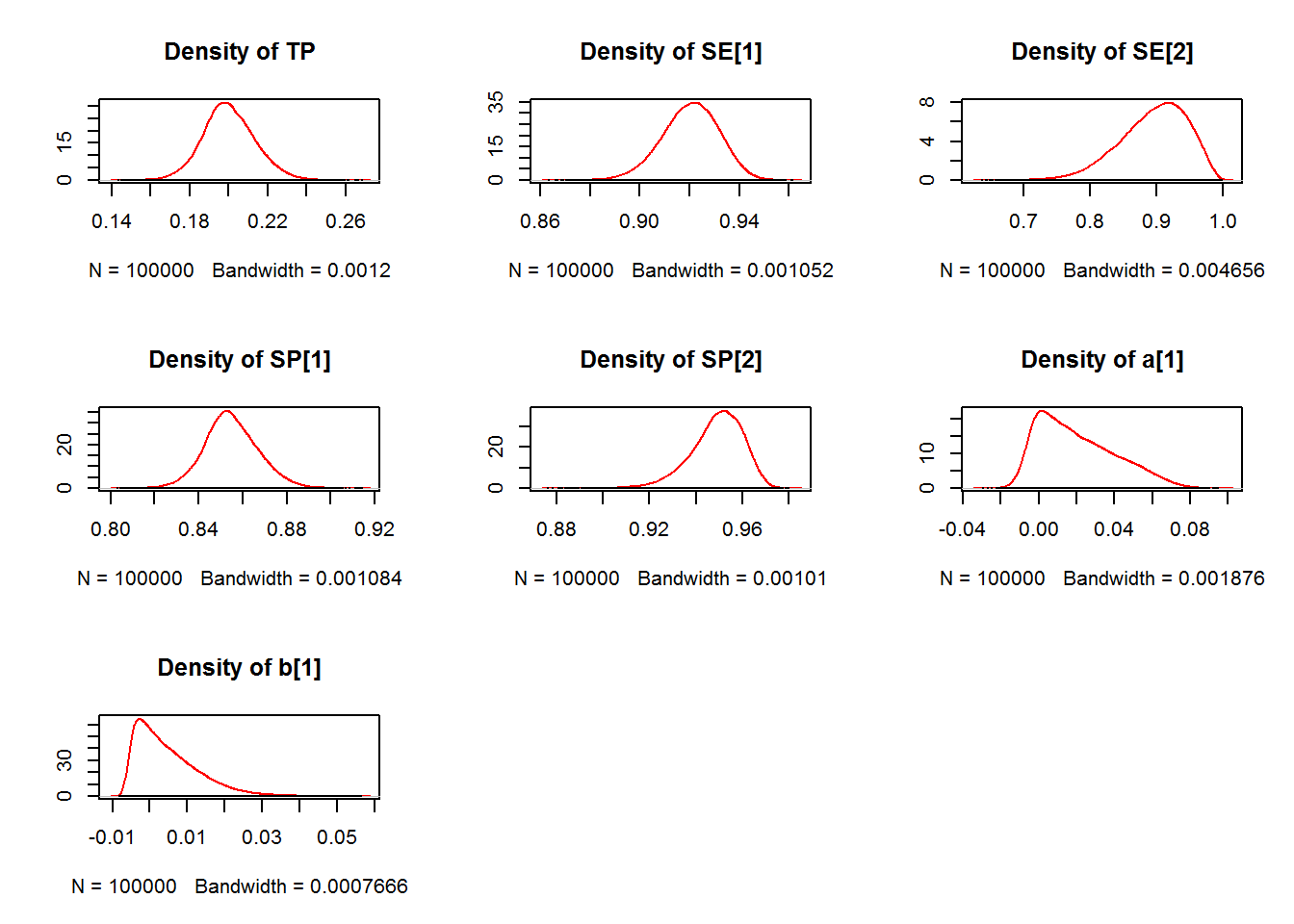


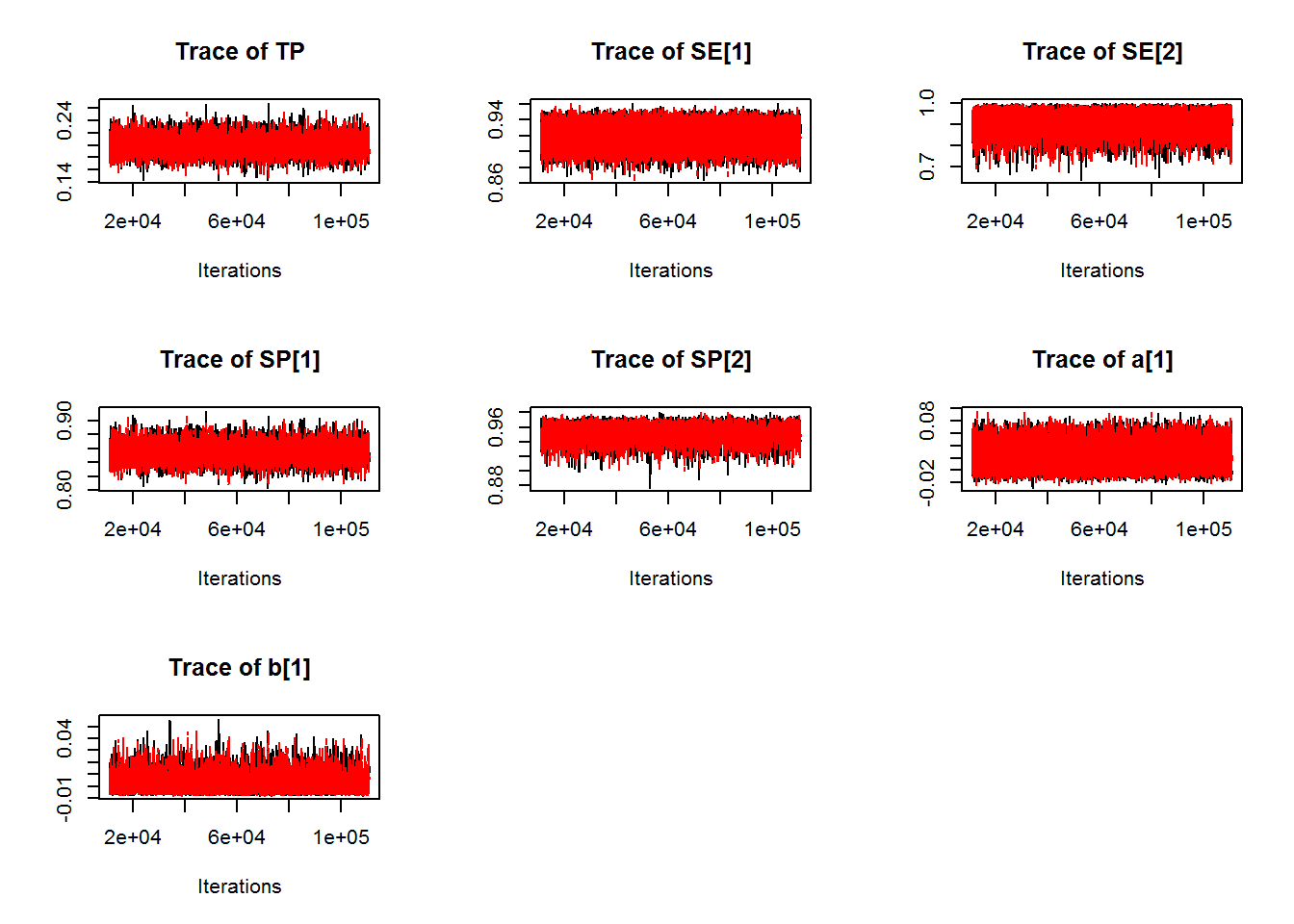


## Uganda

### Summary

## mean median mode sd 2.5% 97.5%

## TP 0.220 0.224 0.232 0.057 0.093 0.321

## SE[1] 0.835 0.842 0.857 0.062 0.693 0.935

## SE[2] 0.610 0.608 0.598 0.103 0.412 0.811

## SP[1] 0.857 0.858 0.862 0.049 0.756 0.948

## SP[2] 0.932 0.938 0.961 0.036 0.850 0.984

## a[1] 0.044 0.041 0.032 0.047 -0.036 0.144

## b[1] 0.041 0.038 0.021 0.028 0.000 0.101

##

## Multivariate BGR statistic = 1.014

## BGR values substantially above 1 indicate lack of convergence

### Diagnostics

## $DIC

## Mean deviance: 48.03

## penalty 2.992

## Penalized deviance: 51.02

##

## $BGR

## Potential scale reduction factors:

##

## Point est. Upper C.I.

## SE[1] 1.01 1.04

## SE[2] 1.00 1.00

## SP[1] 1.01 1.01

## SP[2] 1.00 1.00

## TP 1.00 1.00

## a 1.01 1.04

## b 1.00 1.00

##

## Multivariate psrf

##

## 1.01


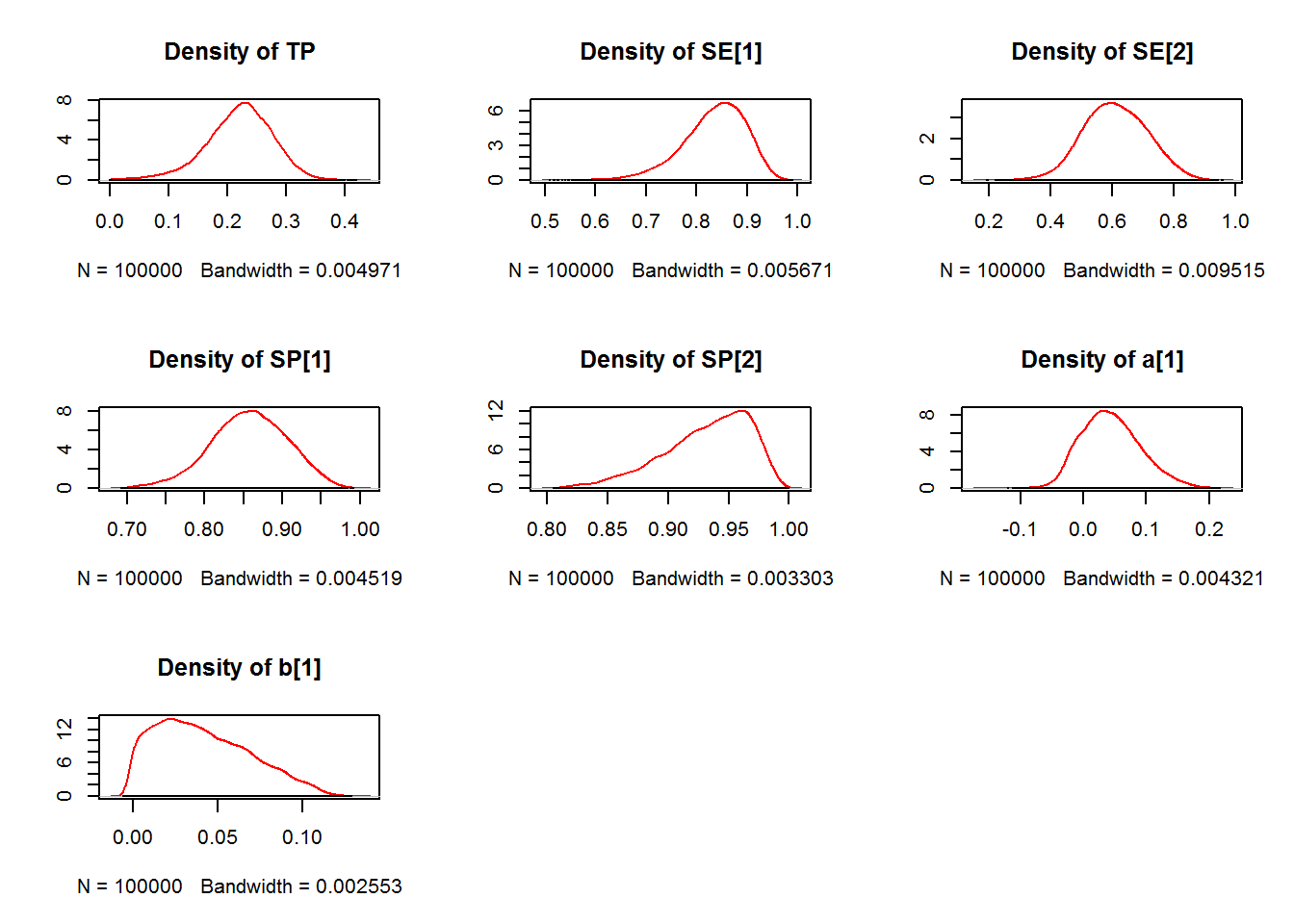


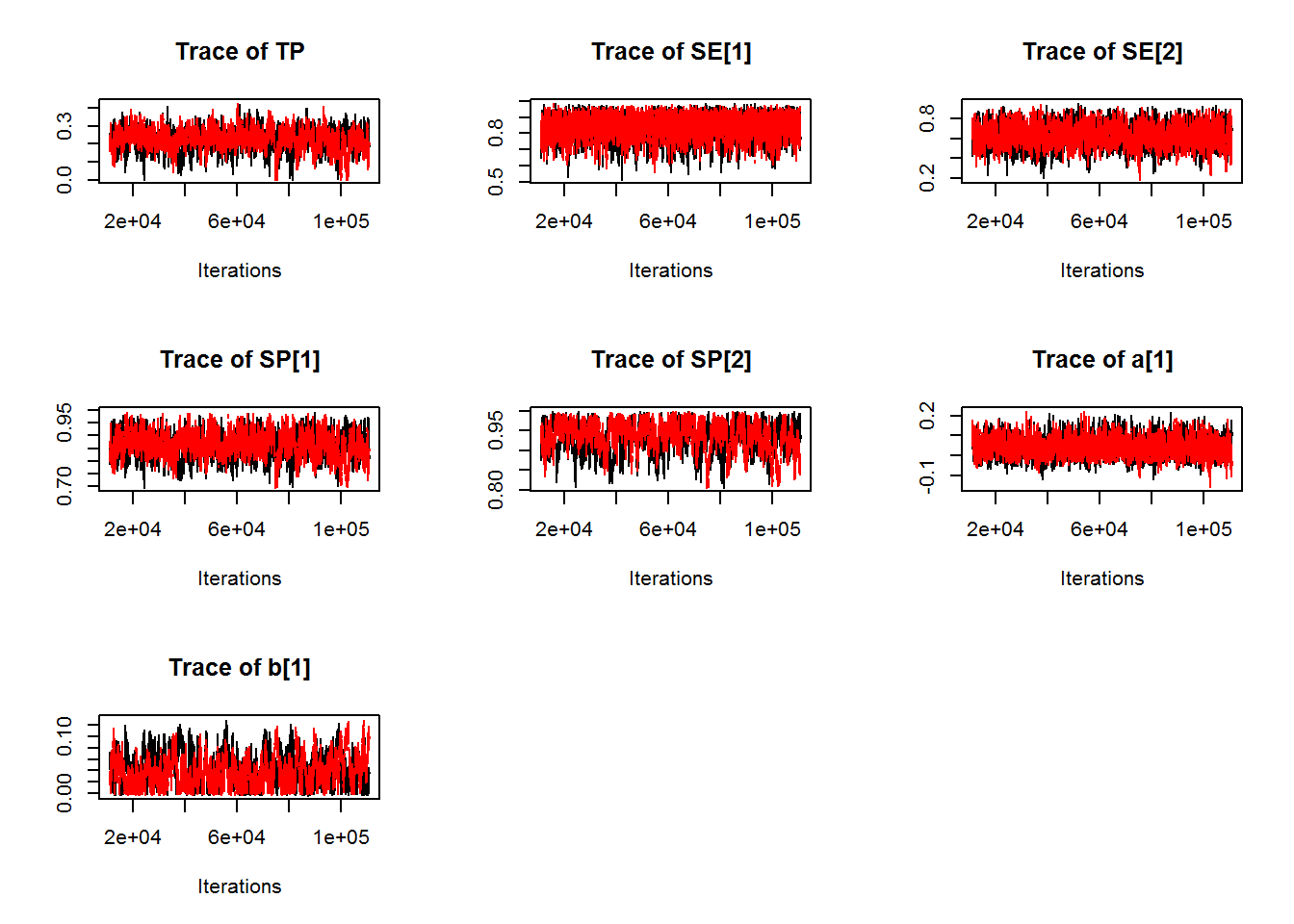


## Kenya

### Summary

## mean median mode sd 2.5% 97.5%

## TP 0.010 0.007 0.001 0.008 0.000 0.030

## SE[1] 0.783 0.788 0.796 0.069 0.634 0.901

## SE[2] 0.771 0.776 0.783 0.067 0.626 0.888

## SP[1] 0.923 0.922 0.921 0.007 0.912 0.939

## SP[2] 0.962 0.960 0.958 0.007 0.952 0.977

## a[1] 0.044 0.042 0.000 0.057 -0.055 0.154

## b[1] 0.029 0.030 0.031 0.005 0.017 0.036

##

## Multivariate BGR statistic = 1.0001

## BGR values substantially above 1 indicate lack of convergence

### Diagnostics

## $DIC

## Mean deviance: 38.56

## penalty 3.023

## Penalized deviance: 41.58

##

## $BGR

## Potential scale reduction factors:

##

## Point est. Upper C.I.

## SE[1] 1 1

## SE[2] 1 1

## SP[1] 1 1

## SP[2] 1 1

## TP 1 1

## a 1 1

## b 1 1

##

## Multivariate psrf

##

## 1


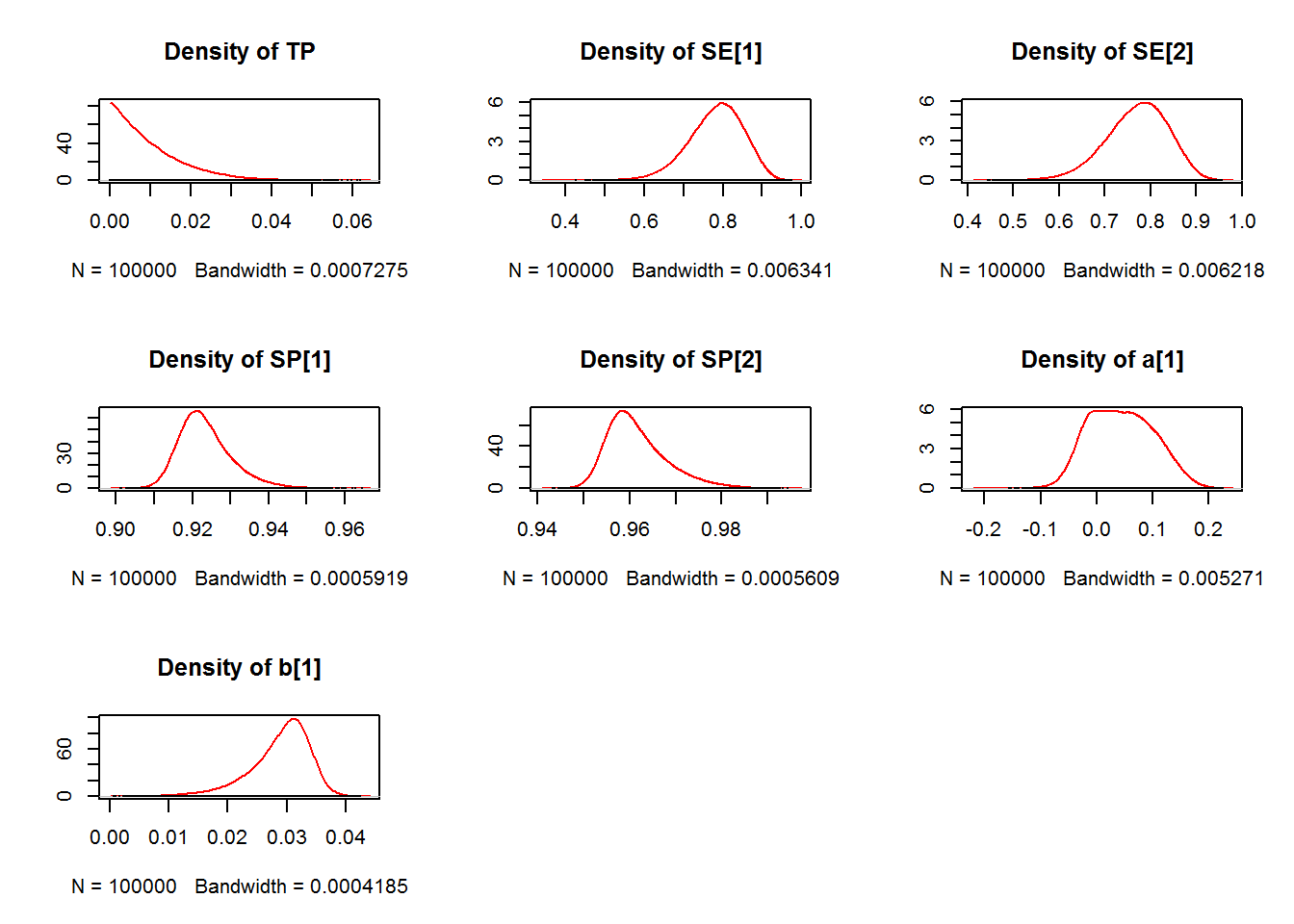

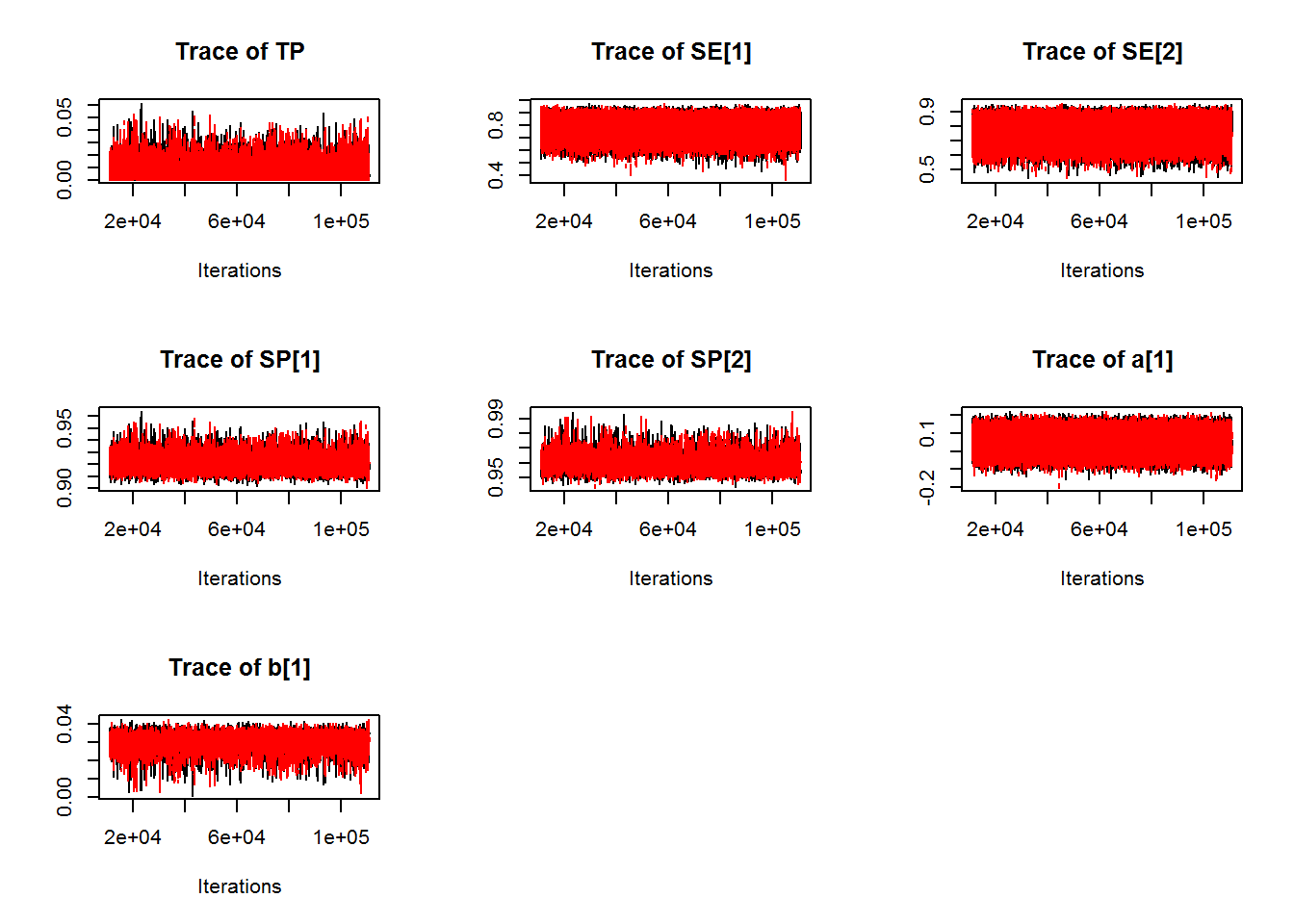

Supplement: Supplementary file 5 — Additional file 5. Detailed output of the 2-test covariance model. [file 12936_2018_2211_MOESM5_ESM.docx]
