# Supplementary material for: True malaria prevalence in children under five: Bayesian estimation using data of malaria household surveys from three sub-Saharan countries
Source: Malar J. 2018 Feb 5;17:65. doi: 10.1186/s12936-018-2211-y (PMC5800038; doi:10.1186/s12936-018-2211-y)
Supplement: Supplementary file 6 — Additional file 6. One-way sensitivity analyses. [file 12936_2018_2211_MOESM6_ESM.docx]

True malaria prevalence in children under five: Bayesian estimation using data of malaria household surveys from three sub-Saharan countries

# Additional file 6. One-way sensitivity analyses.

**Table E1. Estimated true malaria prevalence and diagnostic methods’ sensitivity and specificity by country (mean and 95% uncertainty interval).** The models indicated by an asterisk indicate one-way sensitivity analyses for which the corresponding prior was set to Beta(1, 1).

| **Parameter** | **Full model** | ***RDT, SE** | ***RDT, SP** | ***Microscopy, SE** | ***Microscopy, SP** |
| --- | --- | --- | --- | --- | --- |
| *The Democratic Republic of the Congo* | | | | | |
| True prevalence | 0.200 (0.174–0.228) | 0.204 (0.173–0.243) | 0.142 (0.042–0.206) | 0.220 (0.188–0.249) | 0.202 (0.177–0.229) |
| RDT, SE | 0.920 (0.897–0.941) | 0.906 (0.799–0.985) | 0.919 (0.895­–0.941) | 0.918 (0.894–0.940) | 0.920 (0.896–0.941) |
| RDT, SP | 0.855 (0.832–0.880) | 0.855 (0.831–0.880) | 0.803 (0.726–0.858) | 0.874 (0.844–0.900) | 0.856 (0.834–0.881) |
| Microscopy, SE | 0.896 (0.784–0.975) | 0.892 (0.767–0.977) | 0.929 (0.832–0.986) | 0.648 (0.414–0.875) | 0.899 (0.790–0.977) |
| Microscopy, SP | 0.949 (0.924–0.967) | 0.952 (0.923–0.981) | 0.899 (0.812–0.959) | 0.901 (0.833–0.957) | 0.951 (0.926–0.969) |
| *PSRF* | *1.0023* | *1.0054* | *1.0183* | *1.0227* | *1.0019* |
| *Uganda* | | | | | |
| True prevalence | 0.220 (0.093–0.321) | 0.243 (0.032–0.489) | 0.235 (0.092–0.343) | 0.213 (0.101–0.323) | 0.151 (0.012–0.295) |
| RDT, SE | 0.835 (0.693–0.935) | 0.666 (0.327–0.934) | 0.835 (0.689–0.937) | 0.839 (0.705–0.936) | 0.830 (0.689–0.934) |
| RDT, SP | 0.857 (0.756–0.948) | 0.820 (0.704–0.934) | 0.872 (0.755–0.972) | 0.851 (0.763–0.948) | 0.803 (0.709–0.925) |
| Microscopy, SE | 0.610 (0.412–0.811) | 0.545 (0.302–0.776) | 0.605 (0.433–0.802) | 0.662 (0.371–0.930) | 0.567 (0.331–0.794) |
| Microscopy, SP | 0.932 (0.850–0.984) | 0.923 (0.824–0.985) | 0.940 (0.852–0.987) | 0.937 (0.848–0.987) | 0.883 (0.816–0.972) |
| *PSRF* | *1.0140* | *1.0069* | *1.0123* | *1.0299* | *1.0044* |
| *Kenya* | | | | | |
| True prevalence | 0.010 (0.000–0.030) | 0.008 (0.000–0.023) | 0.014 (0.001–0.039) | 0.012 (0.000–0.038) | 0.014 (0.000–0.041) |
| RDT, SE | 0.783 (0.634–0.901) | 0.510 (0.099–0.905) | 0.787 (0.641–0.903) | 0.777 (0.622–0.900) | 0.783 (0.636–0.900) |
| RDT, SP | 0.923 (0.912–0.939) | 0.920 (0.911–0.933) | 0.928 (0.914–0.948) | 0.925 (0.912–0.945) | 0.927 (0.913–0.948) |
| Microscopy, SE | 0.771 (0.626–0.888) | 0.764 (0.615–0.885) | 0.768 (0.620–0.886) | 0.464 (0.076–0.897) | 0.773 (0.630–0.888) |
| Microscopy, SP | 0.962 (0.952–0.977) | 0.960 (0.952–0.972) | 0.965 (0.953–0.984) | 0.959 (0.951–0.973) | 0.965 (0.953–0.987) |
| *PSRF* | *1.0001* | *1.0017* | *1.0007* | *1.0002* | *1.0002* |

*RDT = Rapid Diagnostic Test; SE = Sensitivity; SP = Specificity*
